# Harnessing Edible Insect Bioactives for Gut Health: A Comprehensive Review on Chitin-Derived Prebiotics and Peptidomic Insights from the Black Soldier Fly

**DOI:** 10.3390/foods14213654

**Published:** 2025-10-27

**Authors:** Thamer Alhasyani, Tarek Ebeid, Mohamed Ghonimy, Saif Alharbi, Mohamed F. Y. Hassan, Abdullah Jarallah, Mohammed Alkhurayji, Ahmed A. H. Abdellatif, Hassan Barakat

**Affiliations:** 1 Central Laboratories—Poultry Health Laboratory Department, Al-Watania Poultry Company, Buraydah 11475, Saudi Arabia; thamr662@gmail.com; 2 Department of Animal and Poultry Production, College of Agriculture and Food, Qassim University, Buraydah 51452, Saudi Arabia; t.ebeid@qu.edu.sa; 3 Department of Agricultural and Biosystems Engineering, College of Agriculture and Food, Qassim University, Buraydah 51452, Saudi Arabia; m.elsayed@qu.edu.sa; 4 Department of Food Science and Human Nutrition, College of Agriculture and Food, Qassim University, Buraydah 51452, Saudi Arabia; 461115970@qu.edu.sa (S.A.); m.hassan@qu.edu.sa (M.F.Y.H.); 461115496@qu.edu.sa (A.J.); 461115947@qu.edu.sa (M.A.); 5 Ministry of Health, Qassim Health Cluster, King Fahd Specialist Hospital, Buraydah 52366, Saudi Arabia; 6 Department of Pharmaceutics, College of Pharmacy, Qassim University, Buraydah 51452, Saudi Arabia; a.abdellatif@qu.edu.sa

**Keywords:** edible insects, bioactives, gut health, prebiotics, peptidomics, black soldier fly, functional foods, sustainability

## Abstract

The growing need for sustainable protein and functional food ingredients has made edible insects stand out as a flexible source of bioactives. Black Soldier Fly larva (BSFL) bioactives, such as chitooligosaccharides (COSs) and peptides, present potential benefits for gut health; nevertheless, their molecular pathways, clinical validation, and commercial scalability have yet to be thoroughly investigated. This study systematically analyzes current progress in BSFL bioactive extraction and characterization, emphasizing enzymatic and thermal processing, controlled enzyme development, and integrated supercritical fluid enzymatic pipelines. We assess preclinical and animal research that illustrates prebiotic modulation of *Bifidobacterium*, *Lactobacillus*, and *Faecalibacterium* populations; antimicrobial peptide-mediated immune signaling; and antioxidant activity. Multi-omics frameworks that connect the microbial metabolism of COS to gut health help us understand how these processes function. A comparison of the regulatory environments for food and feed applications in the EU, North America, and Asia shows that there are gaps in human safety trials, harmonized standards, and techno-economic assessments. Finally, we suggest some next steps: randomized controlled human trials in groups with irritable bowel syndrome (IBS) and metabolic syndrome; standardized data integration pipelines for multi-omics; and life cycle and cost–benefit analyses of modular, vertically integrated BSFL biorefineries with AI-driven reactors, digital twins, and blockchain traceability. Addressing these issues will hasten the conversion of BSFL bioactives into safe, effective, and sustainable functional meals and nutraceuticals.

## 1. Introduction

The global food system is encountering problems it has never experienced before because of population expansion, environmental damage, and limited resources. It means that finding protein sources that do not involve conventionally raising animals is more important than ever. Plant-based proteins, insects, microbes, and cultured meat are all examples of alternative protein sources that could be better for the environment than traditional animal agriculture by more than 80% [1,2]. These choices require a lot less land, water, and feed, and they also release less greenhouse gas [3,4]. Microorganisms are very promising because they do not need a lot of space or changes in the seasons, and they do not use up a lot of carbon with no gas emissions [5]. In fact, insect proteins are an excellent source of food since they have all the amino acids and provide the nutrients your body needs [3]. Nonetheless, significant challenges remain, including manufacturing scalability, regulatory frameworks, customer acceptance, and achieving equivalent nutritional and sensory profiles to conventional products [6,7]. The market for edible insects around the world has expanded yearly by 23% over the previous five years. By 2024, BSFL would make up more than 40% of all insect-meal output [8]. This rise is because BSFL are great at processing feed into protein and bioactive compounds. People have been paying a lot of attention to edible insects as a viable substitute since they better transform food into protein, produce less gas, and need less land than other sources of protein [9]. The black soldier fly (BSF), *Hermetia illucens*, is notable for its ability to bioconvert organic waste into protein- and lipid-rich biomass, providing the twin advantages of waste valorization and nutritional synthesis [10]. Despite these economic enhancements, substantial gaps remain in our comprehension of the mechanisms of BSFL bioactives, their effects on people, and the simultaneous monetization of these elements.

The gut microbiome, a complex bacterial ecosystem, is essential for health by regulating digestion, immunity, and metabolic balance [11]. The composition of this microbial community is influenced by factors such as nutrition, age, lifestyle, and medications. An imbalance in this vital system can result in dysbiosis, which is linked to many health issues [12,13]. Probiotics directly regulate microbiota and immune responses, whereas postbiotics provide bioactive metabolites that maintain microbial homeostasis [14]. Prebiotics selectively stimulate beneficial bacteria and produce short-chain fatty acids (SCFAs) that enhance gut health [15]. Bioactive peptides also show potential as gut microbiota modulators, though their mechanisms remain incompletely understood [16]. These findings extend beyond human health, since similar microbiome-modulating approaches exhibit promise in veterinary contexts [17].

Chitin and bioactive peptides, which come from insects, are interesting compounds that can alter the diversity of gut microbiota and make people healthier. Chitin is what makes up the hard outer shells of insects. It is a component in the production of chitosan and chitooligosaccharides (COSs). These compounds work as prebiotics by selectively promoting beneficial bacteria, including *Bifidobacterium* and *Lactobacillus*, and improving the gut barrier function [18,19]. These chitin derivatives can lower inflammation, kill pathogens, and affect how the immune system works [20,21]. BSFL are particularly advantageous as they generate bioactive peptides released during protein degradation. These peptides can kill germs, protect cells from damage, and change the way the immune system works [21,22]. Chitin from insects has a lot of potential, but it is not used as much as chitin from crustaceans, a way to make bioactive compounds that do not affect the environment [23].

BSF is an excellent model species for sustainable insect farming since it has a lot of desirable traits and can live in a lot of different places. The species reproduces quickly and can turn various types of organic waste into high-quality biomass [24,25]. It has a well-known nutritional profile that includes a lot of protein and a good mix of amino acids, which makes it suitable for both food and feed [26,27]. Recent advances have made it possible to fully explain how to obtain chitin from *H. illucens* biomass. However, extraction is still more complicated than from crustacean sources because protein and chitin interact strongly [28,29]. The species has a complex gut flora that is required for bioconversion to work and could be valuable in biotechnology [30]. These improvements help make insect-based products that are the same for everyone and keep animals safe when they are made in huge quantities [8].

This review aims to (1) contextualize the role of edible insects in addressing global nutrition and sustainability challenges; (2) highlight the importance of gut microbiome modulation for health; (3) examine the biochemical properties and gut-targeted functionalities of insect-derived chitin prebiotics and bioactive peptides; (4) evaluate the BSF as a versatile platform for producing these bioactives; and (5) discuss safety, allergenicity, and regulatory aspects. This study synthesizes current knowledge on chitin valorization and peptidomic profiling in *H. illucens* to identify potential strategies for utilizing edible insect bioactives to improve gut health in both human and animal populations. Essential aspects encompass optimized processing pipelines aimed at yield and purity, mechanistic pathways, comparative regulatory frameworks for food and feed, along with recommendations for clinical trials and life cycle techno-economic assessments.

## 2. Methodology

A systematic literature review was performed to locate and synthesize data on insect-derived chitin prebiotics and peptidomic bioactives, with a focus on *H. illucens*. We used keyword combinations like “edible insects”, “chitin”, “chitosan”, “chitooligosaccharides”, “prebiotic”, “bioactive peptides”, “peptidomics”, “gut microbiota”, and “*H. illucens*” to search five databases (Web of Science, Scopus, PubMed, Google Scholar, and AGRICOLA) for publications from 2015 to 2025. We also did targeted searches and reference screening. Inclusion criteria mandated investigations on insect-derived chitin or peptides exhibiting demonstrated or potential gut-modulating effects via in vitro, in vivo, or in silico methodologies. Exclusions were imposed on non-English papers devoid of methodological specifics, research without functional bioactivity assessments, or entire texts that were not readily accessible. We used an integrative framework to look at eligible articles that looked at (1) methods for extracting and characterizing peptides; (2) functional assays for prebiotic efficacy and peptide bioactivities; and (3) biological context, such as gut microbiota modulation and in silico peptide receptor predictions. Standardized forms were used to collect information about the study’s characteristics, extraction processes, analytical techniques, and bioactivity outcomes. Quantitative data were organized into tables to compare extraction efficiency and bioactivity parameters among species and processing methods. Then, thematic synthesis was performed. *H. illucens* investigations underwent a comprehensive examination of chitin isolation procedures, proteomic processes, and bioactivity correlations to evaluate methodological consistency and pinpoint study deficiencies.

## 3. Edible Insects as Novel Functional Foods

### 3.1. Historical and Cultural Background of Entomophagy

Entomophagy has primary historical roots in Africa, Asia, Latin America, and Oceania, where insects have been devoured for millennia as both dietary staples and culturally significant foods [31,32]. There are more than 2000 edible insect species in the world, spread across 25 different orders. The most commonly eaten groups are Coleoptera (beetles, 31–32%), Lepidoptera (caterpillars and moths, 15–18%), Hymenoptera (ants, bees, and wasps, 14–16%), Orthoptera (grasshoppers, locusts, and crickets, 14%), and Hemiptera (true bugs, 11%). Together, these groups make up about 88% of all edible insect species eaten around the world [33,34]. There are around 1000 kinds of insects in Africa that can be eaten, like ants, crickets, termites, and caterpillars. These insects supply households with limited incomes with vital proteins, fats, and minerals [35,36]. A lot of scholarly articles about edible insects have come out since 2015. Even though people in Western countries do not like the idea, Europe is leading the way in research [37]. Countries that consume insects care more about how they taste and how easy they are to find, while Western countries care more about how healthy they are and how long they last, which makes it easier for people to eat insects [38]. Research indicates that edible insects are more environmentally sustainable, requiring 40–60% less land and water and generating fewer greenhouse gases compared to conventional animals, while providing comparable nutritional advantages [39,40].

### 3.2. Nutritional Composition of Edible Insects

Insects that are safe to eat have perfect nutritional profiles, which makes them a good source of protein that can be grown over time. These insects have 48–70% protein on a dry matter basis and amino acid profiles that are just as comprehensive as those of regular animal proteins [41,42]. Edible insects have a lot of lysine (1.03–8.64 g 100 g^−1^), leucine (up to 10 g 100 g^−1^), and methionine (up to 4.5 g 100 g^−1^). Some species, such as Bombyx mori pupae, have more methionine than soybeans and come close to the levels of egg protein. Protein digestibility tests show that edible insects have protein digestibility-corrected amino acid scores (PDCAAS) between 0.44 and 0.86. These scores are lower than casein’s (0.97) but still similar to those of plant-based proteins. Lysine or sulfur-containing amino acids are usually the limiting amino acids [41,42]. Their lipid content ranges from 21 to 39%, and they have good fatty acid profiles that are high in monounsaturated and polyunsaturated fats, such as omega-3 fatty acids [19,41]. Oleic acid (MUFA), linoleic acid (omega-6 PUFA), and α-linolenic acid (omega-3 PUFA) are the most common fatty acids in most insect species. For example, Tenebrio molitor and Rhynchophorus phoenicis larvae have high MUFA levels, while Acheta spp. have high linoleic acid levels (32.20–41.30 g 100 g^−1^). Orthopterans, like locusts and crickets, store more α-linolenic acid (omega-3) than typical plant oils like sunflower, olive, and soybean oils [42,43]. Insects are excellent sources of micronutrients, and their levels of iron, zinc, and vitamin B12 are usually similar to or higher than those in beef and chicken [44]. For example, edible insects have iron levels that range from 4 to 62 mg 100 g^−1^ of dry matter and zinc levels that range from 9 to 27 mg 100 g^−1^ of dry matter. Some species, such as Oecophylla smaragdina and Odontotermes, are very high in these elements. Insects contain iron and zinc mostly in non-heme forms that are linked to ferritin, transferrin, and other transport proteins. However, there is not much research on how well these minerals are available to people. Edible insects are also a great source of B_complex_ vitamins. For example, dried cricket powder has ten times more vitamin B12 than beef and a lot of riboflavin (0.11–8.9 mg 100 g^−1^), thiamin, and fat-soluble vitamins like A, D_3_, and E [44,45,46]. In addition, insects have special nutrients like chitin-based dietary fiber and bioactive chemicals that fight bacteria and free radicals [47]. The chitin content may provide prebiotic advantages for gut microbiome health [19]. Research indicates that chitin consumption (2–5 g day^−1^ for 3 weeks) fosters beneficial bacterial genera such as Roseburia and Eubacterium, elevates short-chain fatty acid production, including butyrate, and enhances gut microbiota diversity without detrimental effects on overall health, while their resource-efficient production promotes environmental sustainability [3,48].

### 3.3. Overview of Major Bioactive Classes Identified in Insects

Bioactive compounds in edible insects provide health benefits beyond macronutrients. Insect exoskeleton chitin, chitosan, and melanin help gut flora and fight infections like Escherichia coli and Staphylococcus aureus [20,47]. Chitin at 2–5 g day^−1^ promotes beneficial bacterial species like Roseburia and Eubacterium, short-chain fatty acid synthesis, and gut microbiota diversity. Melanin pigments from edible insects like *H. illucens* have photoprotective, immunomodulatory, and stress-protective properties through free radical scavenging processes akin to ascorbic acid [48,49,50]. Edible insect bioactive peptides are a prominent health-promoting class. Antioxidant, anti-inflammatory, antihypertensive, antidiabetic, and immunomodulatory peptides from 12 insect species have been found using peptididomic analysis. Peptides containing 3–20 amino acid residues and molecular weights below 10 kDa are most bioactive. Peptides from Antheraea assama silkworm pupae show ACE-inhibitory IC_50_ values as low as 0.017 μg mL^−1^, surpassing Enalapril at 0.11 μg mL^−1^. Cricket-derived tetrapeptides FVEG and FYDQ modulate cellular antioxidant activity by modulating SOD, CAT, and GSR enzyme systems. Cricket and mealworm protein hydrolysates inhibit reactive oxygen species and modulate cytokines in vitro, with hydrophobic amino acids like tyrosine, phenylalanine, and proline donating electrons to neutralize reactive species [22,51]. Bioactive peptides at 2–5 g day^−1^ improve short-chain fatty acid synthesis, foster Roseburia and Eubacterium, and diversify gut flora. Melanin pigments from edible insects like *H. illucens* have photoprotective, immunomodulatory, and stress-protective benefits equivalent to ascorbic acid due to free radical scavenging [48,49,50]. Peptidomic analysis found 211 immunomodulatory, antioxidant, anti-inflammatory, antihypertensive, and antidiabetic peptides from 12 insect species [52,53]. Cricket and mealworm protein hydrolysates exhibit inhibition of reactive oxygen species and modulation of cytokines in vitro [54]. Phenolic compounds enhance antioxidant capacity and exhibit anti-inflammatory effects [52,53].

Antimicrobial peptides (AMPs) like defensins, cecropins, and lysozymes are also essential bioactives. With 34–51 amino acid residues and six conserved cysteine residues generating three disulfide bridges, insect defensins have broad-spectrum efficacy against Gram-positive pathogens like Staphylococcus aureus. Cecropins kill bacteria and fungi, whereas lysozymes break down bacterial peptidoglycan and alter insect gut immunological signaling [55,56]. Flavonoids and phenols boost antioxidant and anti-inflammatory properties. Plant absorption and endogenous production, such as sclerotization and melanization, provide insects with phenolic chemicals. Edible insects contain protocatechuic acid, 3,4-dihydroxyphenylacetic acid, flavones, flavonols, and anthocyanins, with Tenebrio molitor (mealworm) and Acheta domesticus (cricket) showing high antioxidant activity in DPPH and ABTS radical scavenging assays. Some insect species produce unique flavonoid metabolites not seen in their plant-based diets, which increases antioxidant capability [53,57].

Medium-chain fatty acids, especially lauric acid (C12:0), constitute a distinct class of antimicrobial lipids prevalent in specific edible insects. *H. illucens* (BSFL) possesses significant levels of lauric acid, comprising 40–50% of total fatty acids. Its monoglyceride derivative, glycerol monolaurate (GML), demonstrates extensive antimicrobial efficacy against multidrug-resistant bacteria, enveloped viruses, fungi, and protozoa via mechanisms that disrupt membranes. Lauric acid disrupts bacterial electron transport chains, inhibits membrane-associated enzymes, and effectively addresses microbial resistance via various mechanisms, including the disruption of quorum sensing [58]. These bioactive classes improve microbial balance, barrier function, and inflammation, making insects beneficial diets for chronic disease management and gut health [59].

## 4. Black Soldier Fly (*H. illucens*): Biology, Production, and Safety

### 4.1. Life Cycle and Farming Practices

**Figure 1:**
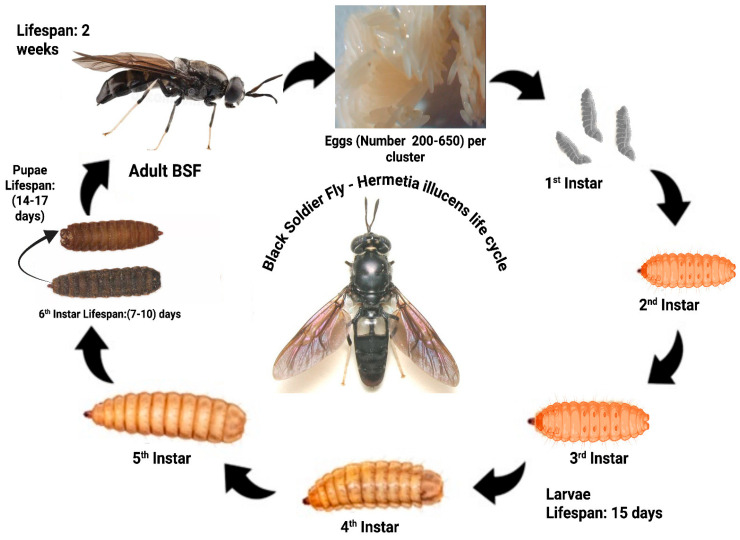
The life cycle of the BSF (*H. illucens*) from egg to adult, illustrating all developmental stages relevant to insect farming and bioactive compound production (created in BioRender BSF life cycle|BioRender, 2025).

BSF develops holometabolously from egg to adult, undergoing biochemical and microbiological alterations (Figure 1). Chitin concentration rises from larval to pupal stages, and recovered chitin is more thermally stable and crystalline than industry standards [60,61]. BSFL is useful for bioactive chemical extraction because larvae accumulate proteins, lipids, and chitin precursors [26]. The bacterial community composition changes during development, with 160 amplicon sequence variations and different metabolic pathways supporting each life phase [62]. Nutrient-rich substrates increase larval growth time, lifespan, and egg production by up to 57% [63]. Adult feeding with carbohydrate supplements like honey significantly enhances female longevity, fecundity, and oviposition success [64]. BSF undergoes complete metamorphosis (Figure 1), with unique developmental phases that affect commercial production and bioactive component extraction. High-quality larval meals like brewer’s grain improve development time, longevity, and egg output [63]. Adults mating many times and females producing multiple eggs boost reproductive output [65]. An adult diet is vital because 5% honey solution boosts female lifespan, fecundity, egg production, and oviposition efficiencies [64]. Gut microbiota vary by substrate and developmental stage, affecting host performance and bioconversion efficiency [30,66]. Chitin and chitosan may be made from breeding failures at different developmental stages [67]. These insects provide sustained proteins, lipids, and bioactive substances [26].

Modern insect farming uses biological features and controlled environments to boost production and sustainability. BSF and mealworm larvae efficiently use brewery leftover grains, fruit and vegetable waste, and manure, lowering feed costs and environmental effects [63,68]. Substrate type, larval density, and moisture content affect larval development, yield, and substrate conversion efficiency, with preferred outcomes employing wasted grains and carefully regulated moisture and density [69,70]. Climate-controlled chambers improve growth and reduce mortality [71]. Late instar harvesting produces homogenous larvae with steady humidity [63]. BSF larval waste makes good organic fertilizer, although its composition and advantages depend on the rearing substrate [72].

### 4.2. Safety Assessment for Human and Animal Consumption

BSFL exhibits considerable promise as an alternative protein source, supported by thorough safety evaluations that address critical concerns. Microbial safety studies indicate that BSFL may contain pathogenic bacteria such as Bacillus cereus, Clostridium perfringens, and Salmonella. However, thermal processing techniques, including blanching and drying, significantly diminish microbial contamination to acceptable levels [73,74]. Assessments of heavy metals indicate cadmium bioaccumulation in larvae; however, concentrations remain within regulatory limits when cultured on compliant substrates [75,76]. BSFL possesses a protein content ranging from 40% to 73%, featuring a balanced composition of essential amino acids and advantageous fatty acid profiles [27,77]. Animal feeding trials indicate favorable growth performance and safety when BSFL substitutes conventional protein sources at suitable inclusion levels [78]. However, substrate quality control and appropriate processing are essential for ensuring product safety [79].

### 4.3. Regulatory Landscape for Edible Insect Use

BSFL manufacturing has regional regulatory and safety issues. Novel foods regulation in the EU requires EFSA risk evaluations and rigorous production criteria for BSFL [80,81]. Despite generating 20,000 tons yearly versus a potential of 3,000,000 tons, insect-based food and feed regulations are lacking in many places, notably in Africa [79]. Multiple linked issues prevent Africa from having adequate insect laws. First, institutional and resource restrictions seriously impede regulation development, including risk assessment technical competence, laboratory capacity, budgetary resources, and inter-ministerial cooperation. Second, 91.7% of African nations lack formal food safety rules due to a lack of scientific data on edible insect safety. Third, authorities prioritize food availability above food safety due to competing food security demands. Fourth, Africa’s fragmented regulatory framework, with ministries managing different areas, produces jurisdictional overlaps and enforcement gaps. Finally, Africa’s low involvement in Codex Alimentarius diminishes its effect on insect safety standards worldwide. These structural impediments contrast with the EU’s centralized regulatory power, significant infrastructure investment, risk assessment protocols, and active international engagement [82,83,84]. Studies show substantial microbial contamination in BSFL, including dangerous bacteria such as B. cereus, *Salmonella* spp., and *E. coli* [74]. Chemical safety issues include potential accumulation of heavy metals and exposure to mycotoxins, pesticides, and pharmaceuticals from substrates [85]. For safe commercialization, the sector needs food safety standards, processing processes, and thorough regulations [86].

## 5. Bioactive Compounds in BSFL

### 5.1. Proteins and Amino Acid Profiles

BSFL is a nutritious alternative protein source. Depending on the raising substrate, BSFL contain 35–50% crude protein by dry matter [87,88]. It contains all necessary amino acids that meet or surpass FAO human nutrition needs, with lysine, leucine, and valine being particularly plentiful [27,87,88]. According to protein efficiency ratio experiments, BSFL protein is equivalent to soybean and fish meal [89]. Protein concentrates can contain up to 73% protein [27,77]. Processing affects nutritional composition. BSFL can replace up to 20% of conventional protein sources in chicken diets without affecting performance [76,78] as a protein supplement for beef cattle [90]. Table 1 shows the essential amino acid content in grams per 100 g compared to beef and soy. BSFL protein amino acid analysis was performed using acid hydrolysis and HPLC, utilizing beef and soy protein values from published literature [91,92,93].

### 5.2. Lipids and Fatty Acid Composition

BSFL has high to moderate lipid content, according to research. Lauric acid (C12:0) dominates the lipid profile, accounting for 36–76% of total fatty acids, depending on rearing substrate [94,95]. Other critical fatty acids include myristic, palmitic, oleic, and linoleic [96,97]. Diet affects fatty acid composition, although BSFL manufacture lauric and myristic acid even without substrate [96,98]. Lauric acid is antibacterial and anti-inflammatory, and BSFL oil may be medicinal [94,99]. The lipid profile’s unsaturated-to-saturated fatty acid ratio depends on substrate composition [100]. These lipids are promising for pharmacological, cosmetic, and feed uses [95,101]. Table 2 summarizes the quantity and physiological functions of BSFL lipids’ main fatty acids.

### 5.3. Chitin and Chitosan: Chemical Structure and Biological Roles

Chitin, a β-(1 → 4)-linked N-acetylglucosamine polymer, is found in insect exoskeletons and may be deacetylated to chitosan, enhancing solubility and bioactivity [102]. BSFL is a potential chitin source, with content varying from 8 to 24% across life stages, peaking in pupal stages, with sheddings affected by biological variables including developmental stage and extraction methods [60,103]. Standard extraction protocols incorporate demineralization, deproteinization, degreasing, and decolorization in order, depending on reagent type, concentration, temperature, and time. Chemical extraction employs acids and bases, whereas biological techniques use enzymatic or microbial fermentation, which affects chitin production and purity differently. Different sample sources (larvae, pupae, shed skins) and post-extraction processing affect chitin content [104,105,106].

BSFL chitin has an α-form structure, good crystallinity (52.8–78.0%), and thermal stability [60,107]. Chitosan and COS are prebiotics that boost gut flora [108]. Cricket chitosan at varied doses boosted probiotic bacteria and inhibited Salmonella typhi [109]. BSFL-derived chitosan also has antibacterial action against different diseases, depending on bacterial species and concentration [110]. These qualities make insect-derived chitin and chitosan useful for dietary, biomedical, and agricultural uses [23]. Table 3 compares the physicochemical features of chitin and chitosan and their gut health effects on solubility, prebiotic effectiveness, and microbiota modification. Chitin generally has ~90% acetylation, while chitosan has ~50%. Chitin is water-insoluble, whereas chitosan is acid-soluble. Chitin’s strong acetylation hinders solubility and fermentation, whereas chitosan’s partial deacetylation improves solubility and selective bacteria fermentability, leading to different gut health functions (Table 3).

### 5.4. Other Bioactives: Peptides, Antimicrobial Agents, Phenolics

**Figure 2:**
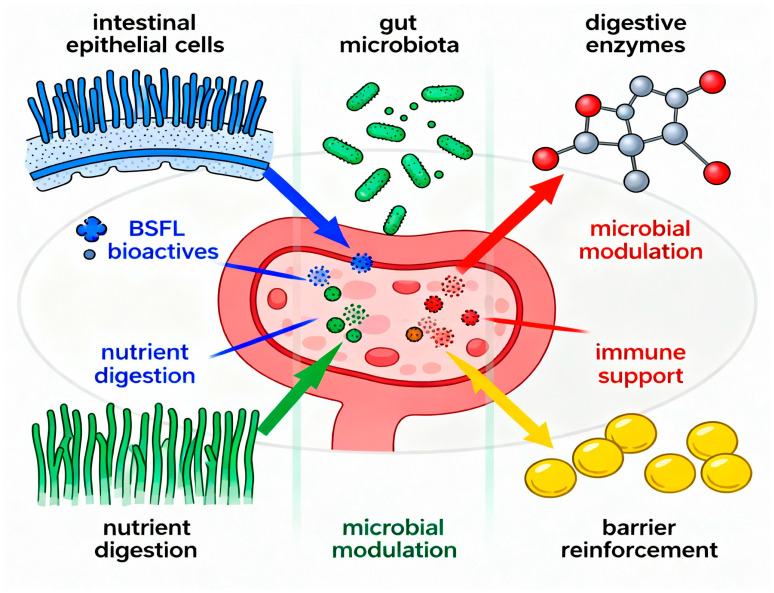
The essential bioactive compounds obtained from BSFL and their diverse functions within the gut ecosystem, such as AMPs, chitin-derived prebiotics, crucial amino acids, and bioactive lipids, which together promote gut health by modulating microbiota, enhancing barrier function, and regulating the immune system.

BSFL generate a variety of bioactive compounds in addition to their main protein and lipid constituents. Proteolytic digestion of BSFL proteins produces AMPs with molecular weights ranging from 0.5 to 3 kDa, exhibiting notable antibacterial activity against pathogenic bacteria [111,112]. These peptides demonstrate antioxidant properties, with particular sequences exhibiting significant radical scavenging activity [113,114]. BSFL also contains phenolic compounds that enhance antioxidant capacity, with an average total phenolic content of 2.5 mg GAE g^−1^, which correlates with DPPH radical scavenging activity [115]. Other bioactive compounds comprise sterols like campesterol and β-sitosterol [116], as well as antimicrobial fatty acid derivatives that function synergistically to inhibit pathogenic bacteria and diminish inflammatory markers [117]. These bioactive compounds collectively enhance immune function and gut health in animal applications [118], as illustrated in Figure 2.

## 6. Chitin-Derived Prebiotics: Mechanisms and Gut Health Benefits

### 6.1. Chemistry and Digestion of Chitin in the Gastrointestinal Tract

**Figure 3:**
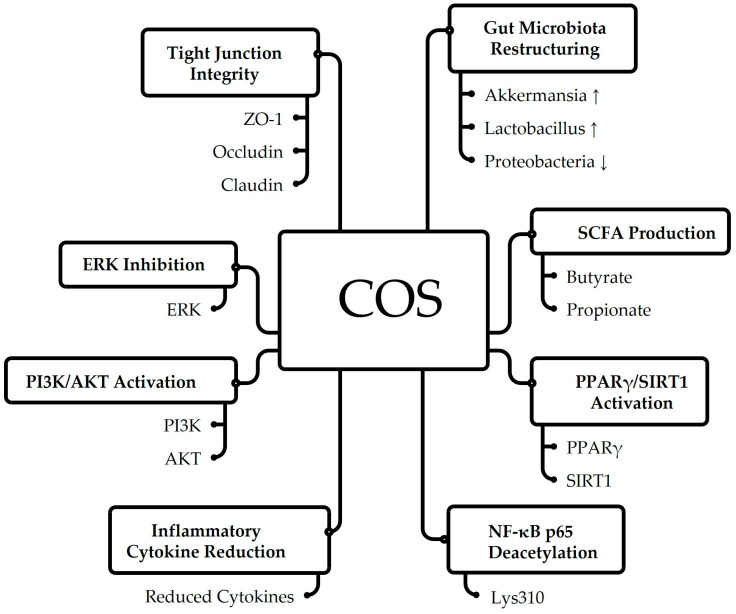
Mechanisms of COS in Modulating Gut Health and Inflammatory Responses.

COS are deacetylated derivatives of chitin with a degree of polymerization less than 20 and a molecular weight below 3.9 kDa, exhibiting enhanced water solubility compared to chitin [119]. These compounds demonstrate significant prebiotic effects by modulating gut microbiota composition, particularly increasing Bacteroidetes while decreasing Proteobacteria and reducing the Firmicutes/Bacteroidetes ratio [120,121]. COS promotes beneficial bacteria, including Bacteroides, Faecalibacterium, and Roseburia, while suppressing pathogenic species like Klebsiella [120,122]. The degree of polymerization influences microbial effects, with COS2-3 enhancing butyrate production and COS4-6 increasing metabolic diversity [123]. Beyond gut health, COS exhibit multiple bioactivities, including anti-inflammatory, antimicrobial, antioxidant, and metabolic regulatory effects [108,119]. Chitin undergoes partial deacetylation in the stomach and microbial fermentation in the colon, producing beneficial metabolites [20,124]. In Figure 3, COS strengthens intestinal barrier integrity through tight junction proteins (ZO-1, occludin, claudin), restructures gut microbiota by increasing beneficial bacteria (Akkermansia, Lactobacillus) while reducing Proteobacteria, and enhances SCFAs production (butyrate, propionate). Additionally, COS activate PPARγ/SIRT1 and PI3K/AKT pathways, promote NF-κB p65 deacetylation, inhibit ERK signaling, and reduce inflammatory cytokines, collectively demonstrating their protective effects on gut health and inflammation regulation.

### 6.2. Role of Chitin and Derivatives as Dietary Fiber and Prebiotics

Chitin, chitosan, and COS are bioactive polysaccharides derived from crustacean exoskeletons, insects, and fungi, demonstrating notable prebiotic and health-enhancing characteristics [20,108]. Chitin serves as an insoluble fiber that enhances fecal bulk and transit time, whereas chitosan functions as a soluble fiber with emulsifying and bile-acid-binding characteristics [108]. COS, which is produced after digestion, specifically stimulates good bacteria like *Bifidobacterium* and *Lactobacillus*, which is a classic example of prebiotic action [18,125]. These molecules demonstrate various biological actions, including anti-inflammatory, antibacterial, antioxidant, and immunomodulatory effects [126,127]. Chitin–glucan complexes derived from mushrooms have enhanced prebiotic effects, markedly elevating propionic and butyric acid concentrations [128]. They can also be used in aquaculture as growth promoters and immunostimulants [129]. They might also be used to make functional foods because they break down and are safe for the environment.

Dietary fibers have different prebiotic effects on gut flora, and the effects depend a lot on the kind, shape, and solubility of the fiber. Numerous studies indicate that the majority of dietary fibers (>80%) are highly fermentable and facilitate the synthesis of SCFAs, especially acetate and butyrate [130]. Fructo-oligosaccharides and inulin are well-known prebiotics that have substantial prebiotic effects. Other fibers, on the other hand, are still being studied as possible prebiotics with different levels of effectiveness [131]. Fibers from cereals, particularly whole grains, reliably elevate bifidobacteria levels and SCFAs synthesis [132]. Comparative studies indicate that fibers may be classified into several categories according to their microbiota-regulating effects, with xylo-oligosaccharides and konjac flour yielding the most significant quantities of SCFAs [133]. Nonetheless, the correlation between fiber supplementation and SCFAs generation in healthy individuals is intricate, with effects varying based on fiber dosage, type, and structural attributes [134,135].

### 6.3. Impact on Gut Microbiota Diversity and Probiotic Growth

Studies indicate that COS and insect-derived chemicals have substantial prebiotic effects on gut flora. COS supplementation alters bacterial composition by elevating beneficial Bacteroidetes and Faecalibacterium, while diminishing harmful Proteobacteria and Klebsiella [120]. In vitro studies demonstrate that COS selectively enhances probiotic development, resulting in up to a 4.1-fold increase in the cell density of Lactobacillus and *C. butyricum* [136]. Chitosan from crickets increases the number of probiotic bacteria while stopping the growth of *S. typhi* [109]. The addition of insect chitin significantly boosts alpha diversity measurements, such as the Shannon index, and encourages beneficial symbionts, including *Ruminococcaceae*, *Lachnospiraceae*, and *Faecalibacterium* [122]. Transplanting fecal microbiota from COS-treated animals enhances intestinal barrier function and reduces cellular apoptosis [137]. These results jointly endorse the prebiotic potential of insect-derived COS and chitosan molecules [108,138].

### 6.4. Effects on SCFAs Production, Gut Barrier Function, and Immune Modulation

SCFAs, especially butyrate, have essential impacts on the immune system and the lining of the gut. Butyrate acts as an anti-inflammatory drug by binding to G-protein coupled receptors GPR41 and GPR43, therefore promoting immunological homeostasis and influencing gene transcription through histone deacetylase (HDAC) inhibition [139,140]. These processes lead to diminished pro-inflammatory cytokines such as TNF-α, IL-1β, and IL-6, while facilitating anti-inflammatory responses [141]. Butyrate improves the strength of the intestinal barrier by increasing the levels of tight junction proteins such as claudin-1 and occludin. This makes the intestines less permeable [142,143]. The molecule is an essential source of energy for colonocytes and helps keep the gut in balance through many different routes [144,145]. These synergistic effects establish butyrate as a potential therapy choice for inflammatory bowel illnesses and other immune-mediated disorders.

### 6.5. Comparative Insights from Animal and Human Studies

Studies on animals and people show that chitin-derived chemicals and insect-based components can have a considerable positive effect on health. In chickens, the addition of chitosan oligosaccharide (COS) to feed at 30–1000 mg kg^−1^ has been found to alter the shape of the intestines by making the villi taller and making the feed conversion more efficient. For example, COS at 30 mg kg^−1^ feed promoted intestinal health and immunity, whereas doses between 200 and 1000 mg kg^−1^ feed have consistently improved growth, antioxidant status, and gut development in broilers [146,147]. COS also lowers inflammatory reactions and boosts the antioxidant capacity of broilers and laying hens [147,148]. In experiments on mammals, adding 5 mg kg^−1^ of COS to their diet made them lose weight, and less fat was built up in their stomachs. Higher dosages, on the other hand, improved metabolic parameters, such as lowering blood glucose and cholesterol levels [39,149]. Components generated from BSFL have immunomodulatory properties via AMPs, lauric acid, and chitin constituents [150]. These substances have prebiotic effects by altering the composition of gut bacteria and improving the function of the intestinal barrier [149]. The research indicates dose-dependent advantages across many animals, underscoring the translational potential of bioactive chemicals produced from insects.

### 6.6. Mechanistic Pathways Linking BSFL-Derived Bioactives to Gut Health

BSFL-derived COS operate as selective prebiotics, mainly aiming at good bacteria in the gut microbiome, such as *Bifidobacterium* and *Lactobacillus* species. Through enzymatic breakdown, these bacteria break down COS and make essential SCFAs, including acetate, propionate, and especially butyrate, through the acetyl-CoA pathway [151]. Butyrate interacts with G-protein-coupled receptors (GPR41, GPR43) on colonocytes, which starts signaling pathways that strengthen the intestinal barrier by increasing the levels of tight junction proteins such as claudin-1 and ZO-1 [152]. At the same time, AMPs from BSFL bind to Toll-like receptors (TLR2, TLR4) on dendritic cells and macrophages, changing NF-κB signaling pathways to lower the production of pro-inflammatory cytokines (TNF-α, IL-6, IL-1β) and raise the levels of anti-inflammatory mediators like IL-10 [153]. This comprehensive molecular framework elucidates the manner in which BSFL bioactives facilitate synchronized interactions between the microbiome and host, hence enhancing gut health and systemic immunological equilibrium. Table 4 gives a short overview of the BSFL bioactives and how they work in microbial, metabolic, receptor, and impact pathways. It shows how BSFL-derived parts are linked to their microbial targets, intermediate intermediates, host receptors that are involved, and impacts that happen later.

## 7. Peptidomics of BSF Proteins

### 7.1. Overview of Peptidomics Technology and Its Application to Insect Proteins

**Figure 4:**
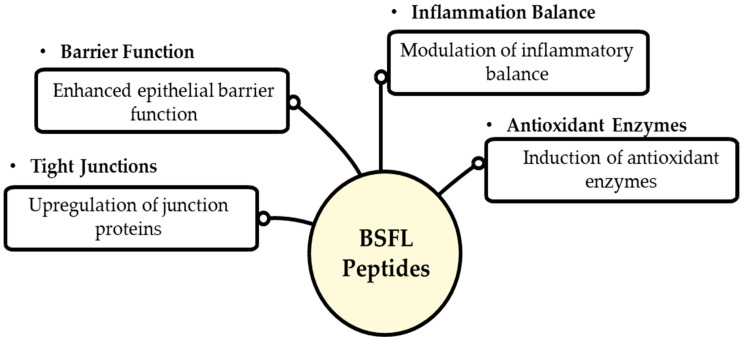
Bioactive functions of BSFL peptides in gut health.

Black Soldier Fly larvae have become a potential source of bioactive peptides that may be used for many different health purposes. Researchers have come up with several ways to extract proteins, and SDS-based buffers give the most protein groups, while other approaches improve proteome coverage [156,157]. Peptidomics methodologies employing LC-MS/MS have elucidated several bioactive peptides derived from BSFL proteins after enzymatic hydrolysis. In silico research on gastrointestinal digestion showed that BSFL proteins can produce peptides that are good for your health, have high GI absorption, and are not harmful. These peptides include antioxidant, anti-ACE, and anti-DPP-IV peptides [114]. Experimental validation has shown that peptides like verified peptides have strong antioxidant properties and can protect HepG2 cells from damage [158]. Peptides produced from BSFL demonstrate anticancer activities via modulating the SKP2/p21/cyclin D1 pathways [112] and cytoprotective effects via Nrf2 ac and exhibit cytoprotective effects through Nrf2 activation [115]. These results emphasize BSFL’s potential as a renewable source of multifunctional bioactive peptides [22]. Figure 4 shows the most critical bioactive activities of peptides made from BSFL. BSFL peptides have four primary positive effects: they improve the function of the epithelial barrier, they increase the levels of tight junction proteins to keep the intestines healthy, they change the balance of inflammation, and they stimulate antioxidant enzymes to protect cells from oxidative stress.

### 7.2. Identification and Characterization of Bioactive Peptides from BSF

Recent studies have shown that bioactive peptides from different protein sources, like BSFL and other edible insects, have a lot of promise. In silico screening coupled with experimental validation has become a proficient methodology for bioactive peptide identification [114,159]. BSFL proteins have potential as sources of health-promoting peptides post-gastrointestinal digestion, with computational analyses indicating antioxidant, ACE-inhibitory, and DPP-IV-inhibitory characteristics [114,116]. Experimental validation has substantiated the anticancer and antioxidant properties of BSFL-derived peptides, with processes involving the modification of SKP2/p21/cyclin D1 pathways [112]. A thorough study revealed 211 potentially bioactive peptides from 12 insect species, with 62 described in vitro and 3 verified in vivo [22]. These findings underscore the increasing significance of insect-derived bioactive peptides for human health applications [158].

### 7.3. Biological Activities of Peptides Relevant to Gut Health

**Figure 5:**
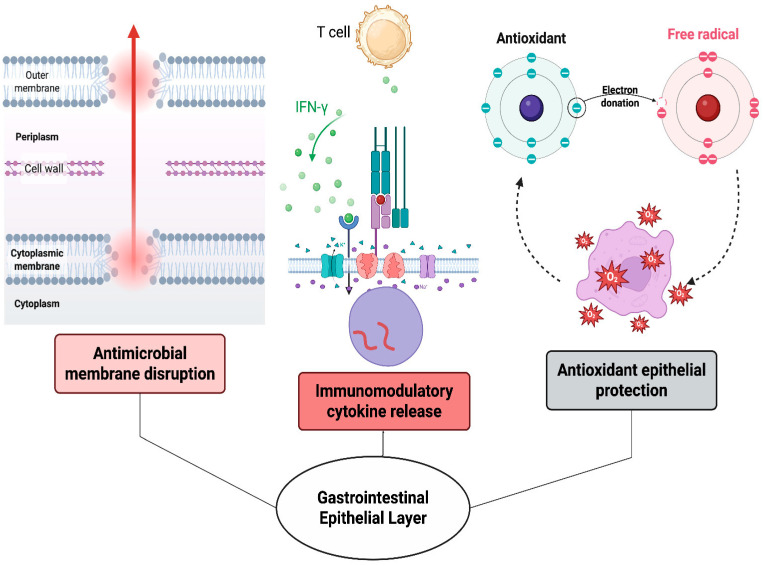
BSF-derived bioactive peptides provide multifaceted protection for gastrointestinal health through coordinated antimicrobial, immunomodulatory, and antioxidant mechanisms as described by Koutsos et al. [166], Created in BioRender GIT protection|(BioRender, 2025).

Bioactive peptides from food show a lot of promise in keeping the gut healthy in a number of ways. These peptides enhance gastrointestinal homeostasis by regulating barrier function, immunological responses, and gut microbiota [160]. AMPs are essential for sustaining tolerance to gut microbiota and safeguarding against enteric infections; alterations in these processes are implicated in the pathophysiology of inflammatory bowel disease [161]. BSF is a promising source of AMPs, with a bioinformatic study identifying 57 putatively active peptides [117]. Certain peptides from diverse origins exhibit anti-inflammatory properties: peptides derived from fermented soybean meal inhibit intestinal inflammation and improve epithelial barrier function [162], whereas peptides sourced from Tricholoma matsutake alleviate colitis by modulating tight junction proteins and inhibiting pro-inflammatory cytokines through NF-κB signaling pathways [163]. These peptides focus on important inflammatory pathways and make the intestinal barrier stronger [164,165]. Figure 5 shows the paths of various peptide activities.

### 7.4. Potential Pathways Through Which Insect-Derived Peptides Influence Gut Microbiota and Host Health

**Figure 6:**
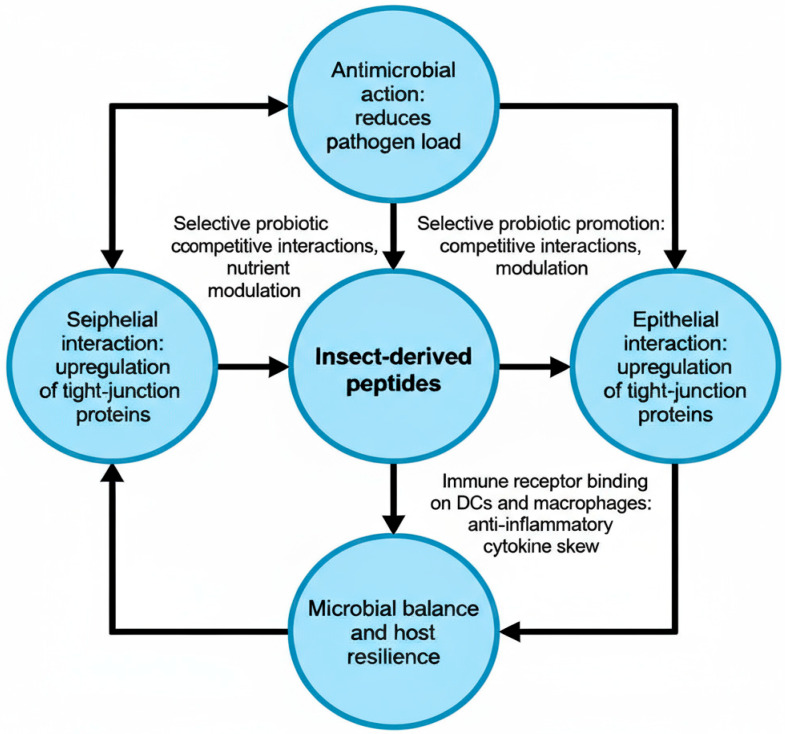
Schematic representation of BSFL-derived bioactive peptides within the gut ecosystem, illustrating their antimicrobial action against pathogens, selective promotion of probiotic microbes through nutrient and competitive modulation, upregulation of epithelial and subepithelial tight junction proteins to reinforce barrier integrity, and immune receptor interactions on dendritic cells and macrophages that skew cytokine profiles toward anti-inflammatory responses, collectively driving microbial balance and host resilience.

Peptides generated from insects have considerable promise in regulating gut ecosystems via several methods. These bioactive peptides demonstrate various activities, including antioxidant, antibacterial, anti-inflammatory, and antihypertensive characteristics [167,168]. Direct antimicrobial action transpires via membrane-disrupting effects on harmful bacteria, whereas advantageous symbionts exhibit enhanced resistance, establishing selection barriers that influence microbial populations [169]. AMPs produced by the gut epithelium facilitate the reorganization of microbiota by selectively attacking pathogens and maintaining commensal bacteria [170]. These peptides help keep the intestinal barrier strong and modulate the immune system. Evidence shows that they help with structural repair and control inflammatory responses [171,172]. The insect intestinal immune system orchestrates many defensive mechanisms, including the synthesis of AMPs via Imd pathways and the formation of reactive oxygen species [173]. Even though there are intriguing uses for functional foods and drugs, more study is needed on safety, rules, and how well consumers take them [168]. Figure 6 shows how the coupled routes help keep the microbial balance and the host’s strength.

## 8. Gut Microbiome Modulation by BSF-Derived Compounds

### 8.1. Interaction Between BSF Bioactives and Gut Microbial Communities

**Figure 7:**
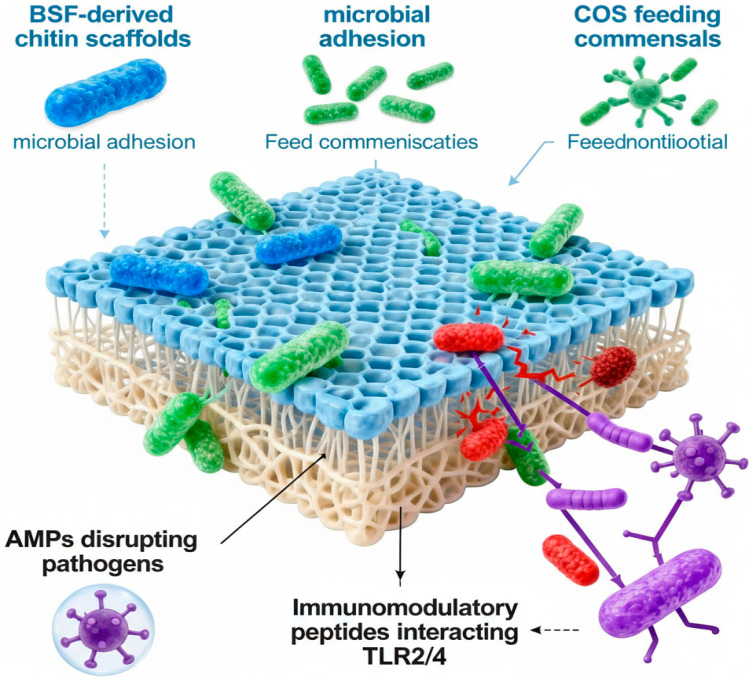
BSF-derived chitin and peptides synergistically shape the gut microbiome: chitin scaffolds support adhesion; COS nourish beneficial commensals; AMPs eliminate pathogens; and immunomodulatory peptides engage TLR2/4 to fine-tune mucosal immunity and promote probiotic resilience.

BSF research has made a lot of progress, and scientists are paying more and more attention to its antibacterial and prebiotic capabilities. AMPs generated from BSF have potent antibacterial properties against a range of pathogens, including Pseudomonas aeruginosa and Staphylococcus aureus, via membrane disruption mechanisms [111,174,175]. The chitin-rich fractions have antibacterial properties, especially against *E. coli*, and they also have prebiotic properties that help good bacteria like Limosilactobacillus reuteri [176]. The gut microbiota of BSF is very important for bioconversion efficiency and substrate processing. Under the right circumstances, beneficial microbes like Lactobacillus and Enterococcus may live in the stomachs of larvae [177,178]. Chitosan compounds from BSF selectively promote good gut bacteria while suppressing pathogens [18]. Future research goals encompass the characterization of microbial populations, the development of genetic resources, and the investigation of BSF’s potential for waste biotransformation and antimicrobial applications [24], Figure 7.

### 8.2. Effects on Microbial Diversity, Pathogen Inhibition, and Host Metabolism

Studies show that microbial diversity greatly improves disease resistance and host metabolism in many ways. Diverse microbiomes safeguard against diseases chiefly by nutrient blocking, wherein communities collectively ingest overlapping nutrients essential for pathogens [179]. Probiotic therapies using Lactobacillus species significantly diminish Salmonella colonization while enhancing gut barrier integrity and immune responses [180]. SCFAs, especially butyrate, are essential microbial metabolites that keep the gut barrier working, provide colonocytes with energy, and change immunological responses both in the gut and throughout the body [144]. Metabolites generated from tryptophan, such as indole-3-propionic acid, have supplementary protective benefits, with supplementation mitigating viral loads and inflammation during influenza illness [181]. Bioactive chemicals in foods like fruits, vegetables, and other foods help good bacteria grow while killing harmful bacteria. This leads to more SCFA synthesis and better metabolic health [182]. These microbial metabolites work together to improve gut homeostasis, barrier integrity, and host immunity [183].

### 8.3. Case Studies in Livestock, Aquaculture, and Experimental Human Models

Studies show that BSFL and its derivatives have a significant effect on gut microbiota and health in many animals. In broiler chickens, the addition of BSF up to 20% had little impact on total cecal microbiota diversity; nevertheless, certain bacterial communities were modified, notably with an enhanced abundance of Roseburia, which may enhance immunity [184]. Replacing soybean oil with BSF oil enhanced the structure of the intestines and changed the populations of microbes in the gut. The best replacement was 50% [150]. BSF-Desmodium meal combinations boosted the number of good lactic acid bacteria in broilers [185]. In Atlantic salmon, the incorporation of BSF meal improved the variety and evenness of the gut microbiota in comparison to traditional proteins [186]. BSF larvae significantly decreased zoonotic pathogens in pig manure via AMPs and gut-associated bacteria [187]. The immunomodulatory capacity transcends diet via AMPs, lauric acid, and chitin components [166]. In laying chickens, live BSF larvae modified particular bacterial species without influencing fear-related behavior [188]. Table 5 highlights the effects of BSF-derived chemicals COS and AMPs on broiler chicks, shrimp aquaculture, and a human pilot study. These results include improved intestinal morphology, a lower pathogen load, and a richer microbiota.

## 9. Technological Considerations and Processing Influences

### 9.1. Methods of Harvesting, Drying, Defatting, and Extracting Bioactive Compounds

Processing BSFL requires a number of stages that are all linked together and have a significant impact on the quality of the final product and the extraction of bioactive compounds. Harvesting happens when the plant is at the late instar stage. Then, it is blanched at 80–90 °C to kill enzymes and lower the number of microbes [190]. Hot air drying (60–70 °C) and freeze-drying are two ways to dry things. Freeze-drying is better at keeping heat-sensitive substances, but it might also cause lipolysis [190,191]. Defatting uses solvent extraction, supercritical CO_2_, or mechanical pressing to get rid of up to 80% of the lipid content [190,192]. Alkaline pH-shifting (pH 10.5–12.5) helps protein extraction by speeding up the process by 135–1080% [193]. Using more than one extraction approach improves proteome coverage [157]. Chitin extraction entails successive deproteinization and demineralization, resulting in 15–25% chitin. However, biological methods utilizing B. subtilis and Acetobacter pasteurianus provide eco-friendly alternatives [28,105]. Combinations of processing methods have a significant effect on the nutritional quality and oxidative stability of the finished product.

### 9.2. Impact of Processing on Stability and Bioavailability of Chitin and Peptides

Processing factors have a significant effect on how stable and bioavailable chitin and peptides from insects are. Maillard reactions can lower peptide solubility and antioxidant activity by up to 30% when drying temperatures are higher than 70 °C [191]. Freeze-drying retains bioactive peptides better than regular heat drying, keeping more than 90% of their bioactivity. However, it costs more in energy and may cause lipid oxidation [194,195,196]. High-temperature treatments lower protein and lipid levels by 17.85% and 22.55%, respectively, while raising chitin levels by 191.21% [191]. Deacetylation conditions impact the quality of chitin. For example, more prolonged exposure to NaOH enhances the degree of deacetylation but lowers the molecular weight [197]. Peptide stability in food matrices is affected by the way they are processed. For example, the way meat is slaughtered, dried, and extracted can all alter the quality of the final product [198,199].

### 9.3. Formulation Strategies for Incorporating BSF Bioactives into Functional Foods and Supplements

BSFL shows a lot of promise as a functional food additive when used in different ways. BSFL protein concentrates are very good at emulsifying, and when heated using ohmic heating, they can make oil-in-water emulsions with interfacial tensions as low as 12.95 mN m^−1^ and droplet sizes as small as 0.68 μm [200]. These protein concentrates have emulsifying activity that is similar to or better than that of whey protein isolate. They can stabilize emulsions with 20–40% oil fractions [201]. Processing techniques have a significant effect on techno-functional qualities. For example, alkaline extraction may make protein concentrates with up to 73.35% protein and improved emulsion stability that can approach 100% [27]. But the way things are processed affects how well they work; for example, blanching makes emulsions less stable and less able to hold together than freezing does [202]. Encapsulation methods provide intriguing strategies for safeguarding BSFL bioactive chemicals, enhancing stability, and facilitating regulated release [203,204]. Even while there are health advantages, there are still problems with safety, consumer acceptability, and following the rules for human ingestion [205,206].

### 9.4. Economic Viability and Regional Industrialization Strategies

Choosing a processing technique for BSFL bioactive extraction requires finding a balance between energy use, bioactivity preservation, and cost-effectiveness. Hot air drying (60–70 °C) is the best option for areas with limited resources since it costs the least to set up ($50,000–100,000), uses a small amount of energy (1.5–2.5 kWh kg^−1^), and keeps 60–70% of the peptides [207,208]. On the other hand, freeze-drying keeps bioactivity better (>90% peptides and chitin), but it uses 4 to 10 times more energy (5 to 10 kWh kg^−1^) and costs a lot of money to set up ($500 K–2 M); thus, it can only be used in developed markets [28]. Enzymatic extraction is a good way to go because it uses 0.5–1.2 kWh kg^−1^ of energy and keeps 85–95% of the bioactivity at moderate capital expenditures ($200–500 K^−1^) [209]. Hot air-enzymatic processing together is best for developing areas. It uses 2–3 kWh kg^−1^ of energy, keeps 75–85% of the bioactivity, and costs $150,000 to $350,000 to set up [105].

To sell BSFL bioactives, you need to make plans that take into consideration the amount of money and energy infrastructure in each area. Regions with a lot of resources may build vertically integrated biorefineries that use freeze-drying and supercritical CO_2_ extraction to make pharmaceutical-grade compounds for $5000–7000 per ton of dried larval meal. Middle-income areas can use modular facilities that combine hot air drying with selective enzymatic extraction for $2500 to $4000 per tonne. The climatic benefits mean that heating needs are cut by 30% to 40% [210]. Low-resource areas use solar-assisted drying in decentralized facilities that cost $1200 to $2000 per tonne. This is competitive with local proteins and helps with waste management. Labor (40–67%), substrate procurement (20–35%), energy (10–25%), and capital depreciation (10–20%) are the main costs of production at all levels. Co-product valorization makes up for 15–30% of these costs [211]. Additionally, an in-depth evaluation of key BSFL bioactive extraction techniques, emphasizing their cost efficiency, energy requirements, and ability to preserve bioactivity, to inform decisions on large-scale industrial applications, Table 6.

## 10. Safety, Allergenicity, and Regulatory Aspects

### 10.1. Microbial, Chemical, and Allergenic Risks in Edible Insect Consumption

Edible insects pose notable microbial, chemical, and allergenic risks that necessitate thorough evaluation. Microbial contamination encompasses pathogenic bacteria, including Bacillus cereus. Processing methods such as blanching can achieve reductions of 3 logs in bacterial counts [212]. Studies have reported no detection of Salmonella or *E. coli* in retail samples [213]. Chemical hazards include heavy metals such as arsenic, cadmium, lead, and mercury, with detection rates of 100%, 79%, 58%, and 74%, respectively, while concentrations stayed within safe limits [213]. Pesticide residues are present, with glyphosate as the predominant compound [213]. Allergenic risks primarily arise from cross-reactive proteins, specifically tropomyosin and arginine kinase, which may elicit reactions in individuals with allergies to crustaceans and dust mites [212,214,215]. Enzymatic hydrolysis, particularly when combined with thermal treatment, has demonstrated a reduction in IgE-binding capacity by 50–60%. For instance, Alcalase hydrolysis followed by heating at 90 °C for 10 min resulted in a 56.3% decrease in IgE binding [216].

### 10.2. Risk Mitigation Strategies and Quality Control Methods

To keep edible insects safe, Good Manufacturing Practices and HACCP frameworks must include all types of biological, chemical, and physical threats [217,218]. Critical control points encompass substrate verification to avert contaminant buildup and microbial decrease by heat treatments, with blanching yielding 3–3.4 log reductions in vegetative bacteria [212]. Heavy metals can build up in insects; therefore, they need to be monitored by inductively coupled plasma mass spectrometry. However, the quantities that are found are usually below legal limits [213,219]. ELISA tests can find cross-reactive allergens such as tropomyosin and arginine kinase [212]. Decontamination stages, including blanching, drying, and new technologies like high-pressure processing for microbial control, are also part of processing pathways [220,221]. Some important pathogens include *S. aureus*, *Clostridium* spp., and the *B. cereus* group. Salmonella and *E. coli* are usually not found in items that have been appropriately treated [213,222].

### 10.3. Current and Evolving Regulatory Frameworks Globally for Edible Insect Products

Current regulations on edible insects differ significantly from one country to the next. Regulation (EU) 2015/2283 governs novel foods for human use and does not yet include BSFL. Regulation (EU) No 2001/999 (Annex IV), as amended by Regulation (EU) 2017/893 (Annex X) [81,84]. Allows insect proteins from seven species to be used in aquaculture, poultry, and swine feed. The most current EU law on animal by-products (2021/1925) allowed the use of processed animal proteins (PAPs) from silkworms (Bombyx mori) in aquaculture, poultry, and pig feed. This added one more species to the list of seven that were already allowed [223]. The FDA uses GRAS findings to check BSFL substances, which means they need a lot of safety information. Countries that traditionally eat insects, like Thailand and Japan, control them using regular food safety rules instead of giving them a new food label [84]. Microbial contamination, heavy metals, and cross-reactive allergens are some of the most critical safety issues. Processing procedures like blanching can significantly lower microbial loads [212]. The rules are changing quickly, and Codex Alimentarius is working on making global standards that are the same for everyone [84]. But regulatory loopholes are still a big problem, especially in poor nations where there are no uniform laws [224,225]. Consumer acceptance varies considerably based on geographical and cultural factors [226].

## 11. Remarks and Future Perspectives

Engineered chitinases and deacetylases tailored through directed evolution, membrane reactors with immobilized proteases, and integrated supercritical fluid extraction and enzymatic digestion pipelines can increase yields, purity, and sustainability of BSFL-derived oligosaccharides and peptides, but reaction kinetics, enzyme stability, and downstream integration are understudied. Multi-omics frameworks that combine genome-scale metabolic modeling with metaproteomic, peptidomic, and metabolomic profiling can reveal the biosynthetic pathways and host–microbe interactions that underlie BSFL bioactive functions. These discoveries must be translated into tailored microbiome modulators using standardized data integration workflows and causal validation studies. Preclinical evidence supports COS and peptide supplements’ gut health potential, but randomized, controlled human trials, especially in IBS and metabolic syndrome cohorts, are needed to define dose–response relationships, mechanistic biomarkers, and sensory acceptability. Finally, techno-economic and life cycle assessments of modular, vertically integrated BSFL production systems with automated climate control, frass valorization, and alignment with waste treatment facilities, policy frameworks, and public–private partnerships will de-risk infrastructure investments and ensure environmental and economic viability for functional food and nutraceutical markets.

In the coming decade, combined developments will alter BSFL-derived bioactive manufacturing. AI-guided evolution will produce more stable and specialized chitinases and deacetylases. Reactors using machine learning will optimize reaction kinetics live. Continuous, solvent-free oligosaccharide and peptide recovery is possible with hybrid ceramic–polymer membrane reactors. Modular supercritical fluid extraction with enzymatic pretreatment increases energy efficiency. Precision fermentation and engineered gut microbiomes will boost chitin production and waste-to-bioactive conversion. Multi-omics platforms will predict and control metabolic flux and chemical buildup in real time. Vertically integrated biorefineries provide large-scale, sustainable output. Blockchain traceability and digital twins provide quality and compliance. These developments will enable insect-derived functional ingredient clinical and regulatory approval.

## 12. Conclusions

Bioactives derived from edible insects, specifically BSFL-derived COS and peptides, exhibit significant prebiotic, antimicrobial, antioxidant, and immunomodulatory properties that contribute to gut health. Advancements in extraction technologies, protein engineering, and integrated bioprocessing offer potential enhancements in product quality and sustainability. Comprehensive clinical trials, standardized regulatory frameworks, and techno-economic evaluations are essential for translating these findings into safe, effective, and commercially viable functional foods and nutraceuticals. The effective utilization of BSFL bioactives relies on the integration of directed enzyme evolution, precision fermentation, real-time multi-omics monitoring, and life cycle-driven production models to produce targeted and sustainable microbiome modulators for both human and animal health.

## Figures and Tables

**Table 1:** Amino acid composition (g 100 g^−1^ protein) of BSFL protein analyzed by acid hydrolysis and HPLC, compared with beef and soy.

Amino Acids	BSFL	Beef	Soy
Lysine	6.2	6.0	5.7
Leucine	8.5	7.9	7.0
Valine	5.7	5.4	4.8
Methionine	1.9	1.6	1.3
Cysteine	1.1	0.4	0.1
Threonine	4.3	4.1	3.8
Phenylalanine	4.7	4.6	4.5
Tryptophan	1.3	1.2	1.1

**Table 2:** Major fatty acids in BSFL lipid fraction (percentage of total fatty acids).

Fatty Acid	Abundance (%)	Health Implications
Lauric acid	36	Antimicrobial, supports gut barrier
Palmitic acid	12	Energy source, structural lipid component
Oleic acid	28	Anti-inflammatory, cardioprotective
Linoleic acid	18	Essential omega-6, supports cell signaling
Stearic acid	6	Neutral effect on serum cholesterol

**Table 3:** Summary of chitin/chitosan’s physicochemical properties and gut health benefits.

Property	Chitin	Chitosan	Gut Health Role
Degree of acetylation	~90%	~50%	Determines solubility and fermentability
Molecular weight (kDa)	100–200	50–100	Influences prebiotic efficacy
Solubility	Insoluble in water	Soluble in acidic solutions	Enables selective microbial fermentation
Biological activity	Structural support	Antimicrobial, prebiotic	Modulates microbiota and strengthens the mucosal barrier

**Table 4:** BSFL bioactives to microbial, metabolic, receptor, and effect pathways.

BSFL Compounds	Microbial Target	Metabolites	Host Receptor	Effect	Ref.
BSF-COS	*F. prausnitzii*	Butrate	CPR109A	Enhanced barrier	[105]
BSF-AMPI	Lactobacillus	Lactate	TLR2	Reduced TNF-α	[154]
BSF-CPP	*B. lungum*	Acetate	GPR43	Anti-inflammatory	[155]

BSF-COS: BSF–chitosan oligosaccharides, BSF-AMPI: BSF–antimicrobial peptide I, BSF-CPP: BSF-derived cell-penetrating peptides.

**Table 5:** Outcomes of BSF-Derived Compound Supplementation Across Models [117,189].

Model	BSF-Derived Compound	Key Outcomes
Broiler chickens	5% COS	20% increase in villus-to-crypt ratio; significant reduction in cecal Enterobacteriaceae counts
Shrimp aquaculture	AMP-enriched BSF protein hydrolysate	1.5 log reduction in *Vibrio* spp. loads; 15% increase in survival rate
Pilot human trial	4 g day^−1^ COS for six weeks	30% increase in stool *Bifidobacterium* abundance; improved bowel regularity

**Table 6:** A comprehensive comparison of primary processing methods for BSFL bioactive extraction, analyzing cost structures, energy consumption, and bioactivity retention to guide industrial implementation decisions.

Processing Method	Energy Consumption (kWh/kg)	Processing Time	Capital Investment	Bioactivity Retention	Operating Costs	Industrial Suitability	Regional Considerations
**Hot Air Drying (60–70 °C)**	1.5–2.5	18–24 h	Low ($50–100 K for medium scale)	Moderate (60–70% peptide retention)	Low (labor, energy)	Highly established technology	Suitable for all regions; limited climate control needed
**Freeze-Drying**	5–10 (4–10x—higher than HAD)	24–48 h	Very High ($500 K–2 M for industrial units)	Excellent (>90% peptide/chitin retention)	Very High (energy, maintenance)	Limited—niche high-value applications	Most suitable for temperate/cold climates; prohibitive in energy-scarce regions
**Enzymatic Extraction (Chitin/Peptides)**	0.5–1.2 (moderate heating)	4–12 h	Moderate–High ($200–500 K)	Excellent (85–95% targeted bioactives)	Moderate–High (enzyme costs, pH control)	Moderate—requires technical expertise	Viable in regions with enzyme availability and trained personnel
**Supercritical CO_2_ Extraction (Lipids)**	2–4	2–6 h	Very High ($800 K–3 M)	Excellent (>95% lipid quality)	High (CO_2_ pressure maintenance)	Moderate—for high-value lipid products	Requires stable infrastructure; suitable for developed markets
**Combined Hot Air + Enzymatic Processing**	2–3	12–20 h	Moderate ($150–350 K)	Good (75–85% overall retention)	Moderate	Highly balanced cost–benefit	Optimal for developing regions; combines affordability with quality

Energy consumption values based on processing 1 kg dry matter of BSFL, Capital investment estimates for medium-scale facilities (500–1000 kg day^−1^ capacity), Bioactivity retention relative to fresh larvae baseline, Operating costs include labor, energy, consumables, and maintenance.

## Data Availability

No new data were created or analyzed in this study. Data sharing is not applicable to this article.
